# Relationship between Inflammation and Oxidative Stress and Cognitive Decline in the Institutionalized Elderly

**DOI:** 10.1155/2015/804198

**Published:** 2015-03-19

**Authors:** Marília Baierle, Sabrina N. Nascimento, Angela M. Moro, Natália Brucker, Fernando Freitas, Bruna Gauer, Juliano Durgante, Suelen Bordignon, Murilo Zibetti, Clarissa M. Trentini, Marta M. M. F. Duarte, Tilman Grune, Nicolle Breusing, Solange C. Garcia

**Affiliations:** ^1^Laboratory of Toxicology (LATOX), Department of Analysis, Pharmacy Faculty, Federal University of Rio Grande do Sul, 90610 000 Porto Alegre, RS, Brazil; ^2^Post-Graduate Program in Pharmaceutical Sciences (PPGCF), Federal University of Rio Grande do Sul, 90610 000 Porto Alegre, RS, Brazil; ^3^Institute of Psychology, Federal University of Rio Grande do Sul (UFRGS), 90035 003 Porto Alegre, RS, Brazil; ^4^Department of Health Sciences, Lutheran University of Brazil, 97020 001 Santa Maria, RS, Brazil; ^5^German Institute of Human Nutrition, 14558 Nuthetal, Germany; ^6^Department of Applied Nutritional Science and Dietetics, Institute of Nutritional Medicine, University of Hohenheim, 70593 Stuttgart, Germany

## Abstract

*Objective*. Cognitive impairment reduces quality of life and is related to vascular and neurodegenerative disorders. However, there is also a close relationship between these diseases and oxidative stress. Thus, the purpose of this study was to assess whether inflammation and oxidative damage are associated with low cognitive performance in the elderly with different housing conditions. *Methods*. The study groups consisted of 32 institutionalized and 25 noninstitutionalized Brazilian elderly subjects. Oxidative damage, inflammation markers, and cognitive function were evaluated. *Results*. The results demonstrated pronounced oxidative stress in the institutionalized elderly group, which also had a lower antioxidant status compared to noninstitutionalized subjects. High levels of proinflammatory cytokines were also observed in the institutionalized elderly. Furthermore, the raised levels of inflammatory markers were correlated with increased oxidative stress, and both were associated with low cognitive performance. However, based on multiple linear regression analysis, oxidative stress appears to be the main factor responsible for the cognitive decline. *Conclusions*. The findings suggest that individuals with lower antioxidant status are more vulnerable to oxidative stress, which is associated with cognitive function, leading to reduced life quality and expectancy.

## 1. Introduction

Aging is a natural and universal phenomenon [1]. With the progressive increase in life expectancy, it is estimated that the number of >60-year-olds will exceed 1 billion people within the next 10 years [2]. This is mainly due to the rate of aging in countries with developing and emerging economies, such as Brazil, where currently two-thirds of the population is 60 years old or more. It is projected that by 2050 about 80% of the elderly will live in the developing world [2].

The process of aging is multifactorial and heterogeneous and cannot be described or explained without taking into account three aspects: the biological, the psychological, and the social aspects [3]. It is known that the institutionalization of the elderly causes changes in their lifestyle and is often accompanied by psychological and social deficiencies due to isolation from familiar surroundings [4, 5]. The very fact of living in a public retirement home leads to a reduction in their autonomy and can result in a reduced quality of life [6].

Moreover, human aging is characterized by increased susceptibility to age-related diseases and, consequently, by the presence of multiple pathologies and comorbidities [1], characterized by chronic processes, such as inflammation [5, 7], which in conjunction with immunosenescence result in a decline of multiple physiological systems, vulnerability, and the fear of functional dependence [8]. There has been increased interest in the role of inflammation in memory and learning deficits, since disorders like Alzheimer's disease are associated with elevated levels of proinflammatory cytokines combined with decreased levels of anti-inflammatory cytokines [9].

There is increasing evidence indicating that oxidative mechanisms also play a pathogenic role in chronic diseases [1, 10]. Although the physiology of aging remains controversial [11], many theories attribute aging [1] to increased oxidative stress and reduced redox status [10, 12]. Oxidative stress is defined as an imbalance between reactive oxygen (ROS) and nitrogen species (RNS) and attenuated antioxidant defenses [3, 12]. Moreover, reduced glutathione (GSH) levels have been found in the elderly [1]. In fact, the human organism is constantly exposed to a large number of ROS and RNS from both physiological and pathophysiological conditions [1, 13, 14]. If these reactive species are not immediately deactivated or removed by antioxidant pathways, they might accumulate in cells [15], damaging lipids, proteins, and DNA [16]. The antioxidant pathways are elaborate defense systems which protect the human organism from oxidative damage, consisting of enzymes such as catalase, superoxide dismutase, glutathione peroxidase, and numerous nonenzymatic antioxidants, endogenous, like GSH, or nutritional, like vitamins A, C, and E, and carotenoids [5, 17].

Therefore, the accumulation of damage caused by oxidative stress, like oxidized proteins, glycated products, and lipid peroxidation leads to degeneration of neurons, generally found in brain disorders [16]. Cerebrovascular diseases, in turn, are characterized by vascular lesions and recognized as a reason of cognitive decline and dementia in old age [18]. Additionally, in brain tissue, ROS are generated by microglia and astrocytes and seem to modulate synaptic and nonsynaptic communication between neurons and glia and may lead to neuroinflammation and cell death, triggering neurodegeneration and memory loss [16]. Considering that cognitive abilities are crucial for maintaining life quality during the aging process [8], the aim of this study was to investigate the association between inflammation and oxidative status and cognitive performance, evaluated by Minimental Status Examination (MMSE), Verbal Fluency, and Boston Naming Test, in the institutionalized and noninstitutionalized elderly from South Brazil.

## 2. Methods

### 2.1. Study Population

This study was approved by the Ethics Committee of the Federal University of Rio Grande do Sul (number 15146) and the Ethics Committee of the Clinical Hospital of Porto Alegre (number 110171). All volunteers provided their written informed consent.

Eighty elderly subjects were recruited to participate in this study. Among them, forty were institutionalized in different philanthropic nursing homes, and forty were noninstitutionalized elderly subjects from a primary care unit. All subjects lived in Porto Alegre, Brazil.

Individuals were excluded if they had levels of vitamin B12 below normal. Moreover, subjects with cancer, congenital neurological or psychiatric disorders, advanced neurological diseases with difficultly in verbal communication, and fully or partially removed stomach and those who relied on parenteral nutrition in the past or used any multivitamins were also excluded from this study. All volunteers were nonsmokers and had no diagnosis of any cognitive problem. Thirty-two institutionalized elderly and twenty-five noninstitutionalized elderly subjects fulfilled our study criteria and participated in the study.

All subjects answered an investigator-administered questionnaire to assess general health, comorbidities, lifestyle, and educational status. A standard evaluation of comorbidities was also performed with the Charlson Comorbidity Index as described by Charlson et al. (1987) [19]. In addition, the functional abilities of the participants were assessed with the Barthel Index, an instrument used to determine a patient's level of independence in basic daily activities based on a large panel of several functional variables as described previously [20].

### 2.2. Sample Collection

Venous blood samples were collected from all subjects after overnight fasting and placed in heparinized tubes, EDTA-containing tubes, and tubes without anticoagulant. For glutathione peroxidase (GPx) enzymatic activity, whole blood was collected with heparin. Serum and plasma-EDTA were obtained by centrifugation at 1500 ×g for 10 minutes at 4°C. In addition, the serum samples were used to determine inflammation markers, vitamin C, and concentrations of high density lipoprotein cholesterol (HDL). To determine carotenoids, retinol, *α*-tocopherol, malondialdehyde (MDA), and protein carbonyls (PCO) levels, EDTA plasma samples were used. For MDA, serum vitamin C, and HDL analyses, samples were processed immediately. The samples were stored at −80°C until analysis. During the analysis, samples were kept on ice and protected from light if necessary.

### 2.3. Oxidative Damage Biomarkers

#### 2.3.1. Plasma MDA

After alkaline hydrolysis, quantification of lipid peroxidation was assessed by analyzing malondialdehyde levels by high performance liquid chromatography (HPLC) with a detector set at 532 nm wavelength (HPLC-VIS), as described previously [21]. MDA levels are expressed as *μ*mol L^−1^.

#### 2.3.2. Protein Carbonyls (PCO)

Protein carbonyls were measured by a sensitive ELISA method according to Buss et al. (1997) [22]. Total protein concentration in plasma was measured by the Bradford method using bovine serum albumin as a standard. PCO levels were determined as follows: plasma samples were diluted with PBS buffer to a normalized concentration of 4 mg protein mL^−1^ and then samples were derivatized with 2,4-dinitrophenylhydrazine (DNPH) and incubated in Maxisorp multiwell plates (Nunc Immuno 96 Microwell Maxisorp) overnight at 4°C in the dark. Protein carbonyls were detected using a dinitrophenyl rabbit IgG-antiserum (Sigma, Deisenhofen, Germany) as the primary antibody and a monoclonal anti-rabbit immunoglobulin G peroxidase conjugate (Sigma) as the secondary antibody. Color development was performed with* o*-phenylenediamine and H_2_O_2_ and the reaction was stopped with H_2_SO_4_ after 15 min incubation at 37°C. The absorbance was measured using a microplate reader (SpectraMax M2, Molecular Devices) with a detection wavelength of 492 nm. Each sample was analyzed in triplicate. Plasma protein carbonyl concentration was expressed as nmol mg^−1^ protein.

### 2.4. Antioxidant Biomarkers

#### 2.4.1. Glutathione Peroxidase Activity (GPx)

The enzymatic antioxidant activity of glutathione peroxidase (GPx) was measured according to the spectrophotometric method described previously [23] and absorbance was monitored at 37°C in a microplate reader (SpectraMax M2, Molecular Devices) at 340 nm for 6 minutes with readings every 20 s. GPx activity was expressed as *μ*mol NADPH min^−1^ mg^−1^ protein.

#### 2.4.2. Exogenous Antioxidants

Simultaneous quantification of lycopene, *β*-carotene, retinol, and *α*-tocopherol was performed as previously described [24]. Plasma samples were extracted with ethanol : n-butanol solution (50 : 50, v/v) and supernatants were injected into the HPLC system. Absorption was monitored at 450 nm for the quantification of lycopene and *β*-carotene. Fluorescence at two different excitation and emission wavelengths was monitored to quantify retinol (340 and 520 nm, exc./em.) and *α*-tocopherol (298 and 328 nm, exc./em.). Results were expressed as *μ*mol L^−1^.

Serum vitamin C was analyzed according to the method of Baierle et al. (2012) [25]. Vitamin C levels were assessed by HPLC with ultraviolet detection (UV) using tris[2-carboxy-ethyl] phosphine hydrochloride (TCEP) as a reducing agent. After deproteinization of the sample with perchloric acid 10% (v/v), the supernatant obtained after centrifugation was injected into the chromatograph [25]. Vitamin C concentrations were expressed as mg L^−1^.

### 2.5. Inflammation Markers

The cytokines quantification was assessed by ELISA using commercial kits (eBIOSCIENCE, San Diego, USA) for human interleukin-1*β* (IL-1*β*), interleukin-6 (IL-6), interleukin-10 (IL-10), tumor necrosis factor-alpha (TNF-*α*), and interferon-gamma (IFN-*γ*), according to the manufacturer's instructions. The results were expressed as pg mL^−1^, except for IFN-*γ* which was expressed as *μ*g mL^−1^.

### 2.6. Cognitive Assessment

Cognitive assessment was carried out by a psychologist through the application of three instruments in individual interviews. A global examination of cognition was made using the Mini-Mental State Exam, MMSE [26, 27], which assesses orientation, memory, attention, language, and spatial abilities, whose score ranges from 0 to 30 points. An assessment of the ability of search and retrieval of data based on long-term memory was made through the Verbal Fluency – Category Animal [28], which requires organizational skills, self-regulation, and working memory. The category fluency is a timed task in which participants are asked to recall as many animals as they can in 60 seconds, generating a score. This test assesses verbal fluency, traditionally seen as a test of language, semantic memory, and executive function. Lastly, Boston Naming Test (short version) [29, 30] was applied. This test is considered a test of language skills. A subject is presented with fifteen figures and if rightly named each response is scored. High scores on all tests denote better performance and the tests chosen are appropriate for this age group and allow discrimination between good and poor performances. However, five subjects refused to participate in this stage and the sample *n* was reduced for this particular assessment.

The three tests were taken from the Brazilian adaptation of CERAD Battery (Consortium to Establish a Registry for Alzheimer's Disease), used to assess symptoms of Alzheimer's disease. The capacity for Alzheimer's disease detection in a Brazilian cohort was investigated by Bertolucci et al. (2001) [29]. The sensitivity and specificity for the MMSE were 97.6% and 75.3%, respectively. Verbal Fluency showed sensitivity of 73.8% and specificity of 87.1% and, for the Boston Naming Test, psychometric parameters were 61.9% and 69.4% regarding sensitivity and specificity, respectively.

### 2.7. Statistical Analyses

The data were analyzed using SPSS (Statistical Package for the Social Sciences, version 18). Data are presented as mean ± standard error of the mean (SEM) for continuous variables. Categorical variables, presented as frequencies (percentages), were compared between groups using Fisher's exact test. Comparisons between elderly groups were achieved by Student's *t*-test and Mann-Whitney *U* test according to variable distribution. Correlation tests were performed according to Pearson's or Spearman's rank following the variables distribution. Linear regression analyses were applied to adjust the influence of age and Charlson Comorbidity Index on inflammation markers, oxidative biomarkers, and cognitive tests. Additionally, multiple regression models were used to identify the relative contribution of oxidative stress and the contribution of inflammation on cognitive performance. The influence of age, educational status, and comorbidities were also considered. Charlson Comorbidity Index incorporates age in the scoring; thus, models that included age as a separate covariate were eliminated. Variables that had nonnormal distribution were log transformed to be included in multivariate regressions. The results of multiple linear regression models were presented as a set of estimated intercept values, standardized *β* coefficients, and *P* values. *P* values less than 0.05 were considered significant for all tests.

## 3. Results

The baseline characteristics and the prevalence of comorbidities in the studied groups of the elderly are presented in Table 1. All the elderly aged 60 years or more; nonetheless the institutionalized elderly group was found to be older than the noninstitutionalized group (*P* < 0.05). Accordingly, all other parameters were compared by adjusting for age. Regarding Barthel Index, the institutionalized elderly showed lower level of functional independence than the noninstitutionalized elderly (*P* < 0.05), although their score was above the cutoff (80 points) that characterizes dependence for basic daily living activities [31]. Furthermore, it has been shown that both elderly groups had comorbidities, such as hypertension, which was the most prevalent, followed by diabetes and dyslipidemia; however, no significant differences were noted between the groups (*P* > 0.05). On the other hand, the Charlson Comorbidity Index, which takes into account comorbidities as well as age, was significantly different (*P* < 0.05) between the two groups.

HDL levels were 44.94 ± 1.70 versus 58.52 ± 3.48 mg dL^−1^ in institutionalized and noninstitutionalized elderly group, respectively (*P* < 0.05). However, both groups presented levels in accordance with the reference value, which is higher than 40 mg dL^−1^ [32].

Oxidative damage biomarkers, such as lipid peroxidation (MDA) and PCO, were higher in the institutionalized elderly group (*P* < 0.01; Table 2). Additionally, these two oxidative biomarkers were positively correlated (*r* = 0.377; *P* < 0.01), while PCO was inversely associated with HDL (*r* = −0.399; *P* < 0.01).

The enzymatic activity of the antioxidant glutathione peroxidase (GPx) was significantly decreased in the institutionalized elderly compared to noninstitutionalized ones (*P* < 0.001; Table 2) and was negatively correlated with PCO (*r* = −0.412; *P* < 0.001) and MDA (*r* = −0.498; *P* < 0.001). Levels of exogenous antioxidants, vitamins, and carotenoids are summarized in Table 3. It should be noted that the institutionalized elderly showed lower levels of lycopene, retinol, *α*-tocopherol (*P* < 0.001), and *β*-carotene (*P* < 0.05) than the noninstitutionalized elderly. No significant difference was observed between the groups for vitamin C (*P* > 0.05). All results were within the reference values for adults [32], except for lycopene and retinol in the noninstitutionalized elderly group, which were above the reference values. Moreover, HDL was positively correlated with lycopene (*r* = 0.466; *P* < 0.01) and vitamin C (*r* = 0.344; *P* < 0.05).

**Figure 1:**
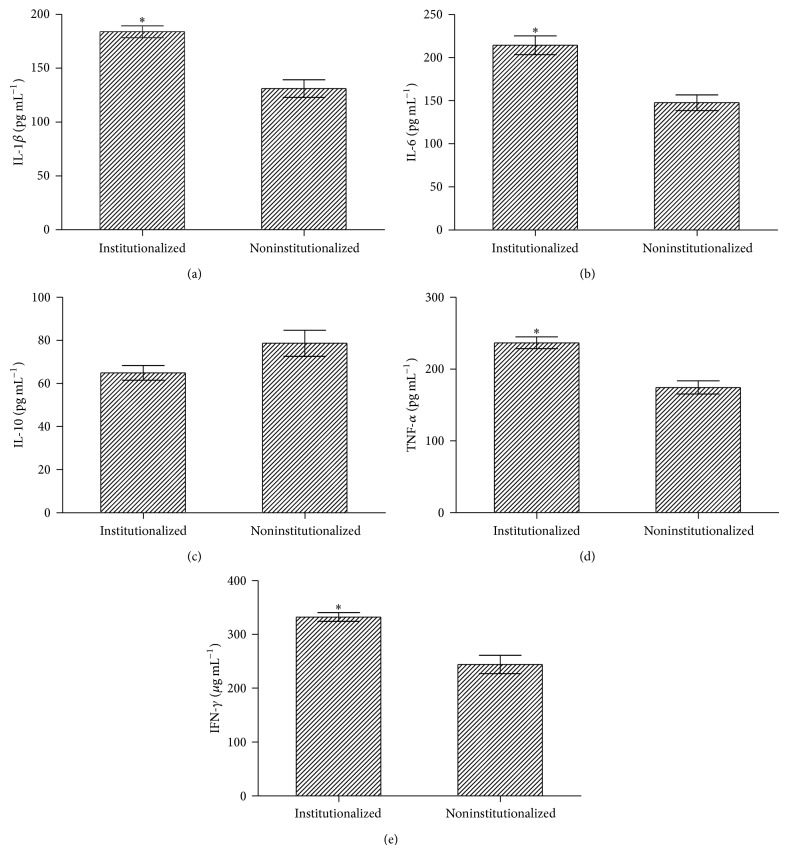
Levels of inflammation markers in the institutionalized (*n* = 32) and noninstitutionalized elderly (*n* = 25). (a) IL-1*β*; (b) IL-6; (c) IL-10; (d) TNF-*α*; and (e) IFN-*γ*. The values were adjusted for age and Charlson Comorbidity Index and expressed as mean ± SEM. ^*^ *P* < 0.001.

The results of inflammation markers of the studied groups are presented in Figure 1. In general, the institutionalized elderly showed higher levels of proinflammatory cytokines (*P* < 0.001) yet no significant difference was observed for IL-10 (*P* > 0.05), an anti-inflammatory cytokine.

Table 4 shows that the high concentrations of IL-1*β*, IL-6, TNF-*α*, and IFN-*γ* were accompanied by high plasma PCO levels and lower GPx activity. In addition, IL-1*β* was inversely correlated with lycopene (*r* = −0.311; *P* < 0.05).

In relation to cognitive performance, significant difference was observed between the elderly groups for the three tests applied, with the institutionalized elderly showing lower scores (*P* < 0.05; Table 5). The institutionalized elderly presented MMSE score below 24 points, the classic cutoff for MMSE [26].

**Figure 2:**
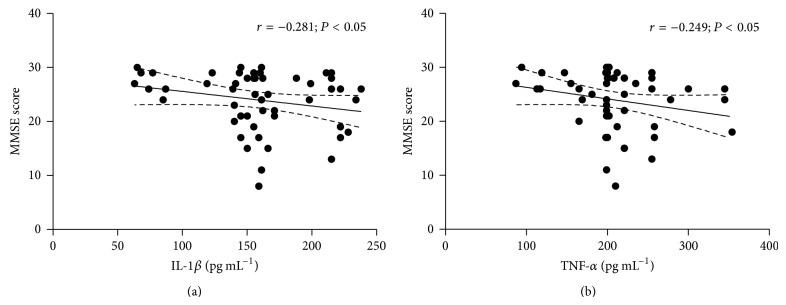
Inflammation markers and cognitive performance. Spearman's correlations among inflammation markers and MMSE performance: (a) IL-1*β* and (b) TNF-*α*. In each analysis, *n* = 52.

The levels of HDL were associated with the cognitive performance in Verbal Fluency (*r* = 0.309; *P* < 0.05) and Boston Naming Test (*r* = 0.396; *P* < 0.01). Moreover, the protein damage, characterized by PCO levels, was negatively correlated with cognitive function, while the GPx activity and the exogenous antioxidant lycopene were positively associated with high cognitive performance (Table 6). Besides oxidative stress, some inflammation markers also correlated with cognition, with the inflammatory cytokines IL-1*β* and TNF-*α* being inversely associated with MMSE (Figure 2).

Additionally, GPx, lycopene, PCO, MDA, and the cytokines TNF-*α* and IL-1*β* were included as independent variables in the multiple linear regressions to explain the cognitive performance. The results of the best-fit models are shown in Table 7 and demonstrated that GPx had a significant influence on the lower cognitive performance in Boston Naming Test. Moreover, only a tendency was observed to GPx in MMSE model, while the educational status was the most significant predictor to this cognitive test, as well as in Verbal Fluency test. The other parameters showed no significant influences on the multiple regression models evaluated.

## 4. Discussion

With the increasing life expectancy in many developed and developing countries, maintaining health in old age has become an important goal, including preventing or optimizing the control of chronic diseases [33]. On the other hand, reference values have not yet been established for endogenous and exogenous antioxidants in the healthy elderly population.

The existence of comorbidities favors the development of nontransmissible chronic diseases. In our study, there was a similar incidence of comorbidities in both elderly groups, supporting the fact that these are the main health problems intrinsic to aging, even in a developing country such as Brazil. It is noteworthy that among the main factors that predispose the elderly to institutionalization are the chronic diseases with their sequelae of inability to perform the basic activities of daily living [34, 35]. Such factors increase with age, supporting the older age and the highest score in Charlson Comorbidity Index observed in the institutionalized group (Table 1). Regarding the social status, individuals who participated in this study, both institutionalized and noninstitutionalized, were considered to be of low economic status based on their income.

The present results showed changes in biomarkers of oxidative damage, resulting in a higher oxidative imbalance in the institutionalized elderly compared to the noninstitutionalized ones. In fact, there is a greater susceptibility to lipid-peroxidative damage at aging, even with adequate diets of exogenous antioxidant [10]. We observed higher levels of MDA in the institutionalized elderly, which is a major product of the reactive species attack on polyunsaturated fatty acids and is widely used as a biomarker of lipid peroxidation [36]. In addition, MDA levels observed in both elderly groups were elevated compared with the levels reported by Roehrs et al. (2011) for healthy adults [37] and in a previous study with the elderly by our group [5]. The brain has high lipid content, second only to the adipose tissue; thus elevated serum levels of lipid peroxidation products have been often reported in brain disorders [16, 38]. In addition, it has been proposed that such products may be promising peripheral biomarkers of underlying white matter abnormalities, given that the axonal membranes and myelin sheaths of the brain are rich in lipids [16].

Regarding the protein damage, the institutionalized elderly had higher levels of protein carbonyls compared to noninstitutionalized subjects. Taking into consideration that the latter group comprises more independent elderly subjects according to the Barthel Index, this finding is corroborative of previous data by de Gonzalo-Calvo et al. (2012) [39], who observed a significant increase in circulating protein carbonyl levels in severely dependent group of the elderly when compared with independent and moderately dependent groups, ranked with the same index [39]. Carbonyl proteins have been used as a global indicator of protein oxidation [1, 5], which is corroborated by the association found between PCO and the other oxidative biomarker, MDA. Oxidative damage to proteins, induced by multiple forms of ROS, has been demonstrated to increase thermodynamic instability and to induce tertiary structural changes that result in inactivation of enzymatic function or protein aggregation, which is a key pathway by which oxidative damage contributes to aging [14, 40, 41].

There is evidence for a reduction of endogenous antioxidants in aging [42]. The important reduction found in the GPx activity of the institutionalized elderly is crucial in the process of ROS neutralization, because this enzyme catalyzes the reduction of hydrogen peroxide (H_2_O_2_) [12, 17]. The modulating activity of this enzyme with age appears to be specific, not only in tissues but also in cellular compartments; for instance, in the heart GPx decreases significantly with age in the cytosol but increases in mitochondria, revealing specific adaptations caused possibly by increased ROS production in mitochondria along the course of aging [12]. In accordance, GPx activity was inversely associated with PCO and MDA, suggesting that a reduction in endogenous defense could favor the increase of oxidative damage, which can lead to enzymatic inactivation in a vicious cycle.

It should be noted that the GPx activity could also be decreased due to lack of its cofactor, selenium. However, in the present study the serum selenium levels were higher in noninstitutionalized than institutionalized elderly groups (*P* < 0.05), yet both were within the reference values (data not shown) [32].

In addition, the levels of exogenous antioxidants were notably lower in the institutionalized elderly than in noninstitutionalized ones. It is known that their concentrations are changed by diet and that hyponutrition occurs frequently in the frailest groups of the population [43, 44]. The elderly, especially those attending nursing homes, are at great risk for certain nutritional deficiencies, as described in Spain [4]. This is especially important in the Brazilian context, where, unlike developed countries, the nursing homes often work under precarious conditions, sources of great preconception with this housing condition. Nevertheless, the levels found here were within the reference values for adults. However, they may differ from those physiologically required for the elderly to a healthy aging.

It is known that oxidative stress has a close relationship with age-related pathologies and consequently with inflammatory processes [45], thus making it difficult to define precisely what triggers the inflammatory response since it involves a large number of different cells and mediators [46]. Cytokines are immune system proteins produced mainly by leukocytes and serve as chemical communicators between cells, regulating host defense against pathogens [47–49]. Significant difference was found in proinflammatory cytokines, IL-1*β*, IL-6, TNF-*α*, and IFN-*γ* between the studied groups, with the institutionalized elderly showing higher levels. In the present work, elevated proinflammatory cytokines levels were accompanied by enhanced PCO levels, confirming the relationship between oxidative damage and inflammatory processes. In agreement, higher proinflammatory cytokines levels were accompanied by reduced GPx activity. Therefore, a close link between inflammation and oxidative stress is recognized, as one activates the other [50]. In corroboration, Campisi et al. (2011) [45] suggest that the accumulation of damaged cells, which increase with age, is implicated in the age-related elevation in circulating inflammatory cytokines, which, in turn, are thought to promote a variety of chronic degenerative diseases [7].

There is strong evidence that the IL-6 pathway is involved in the pathophysiology of chronic diseases often observed in the elderly [51–53]. Hypertension, dyslipidemia, and diabetes are considered vascular risk factors and are associated with both vascular disease and dementia [18]. Many chronic vascular diseases are progressive processes initiated and propagated by local inflammation of large- and medium-sized arteries [54]. It is relevant in this regard that proinflammatory signaling mechanisms in the vascular wall have been well characterized and the risk of developing age-related neurodegenerative disease is associated with increased blood levels of inflammatory cytokines, such as IL-6 and TNF-*α* [8].

It was possible to observe a lower cognitive performance in the institutionalized elderly group compared to the noninstitutionalized one, evidenced by the significant difference in the cognitive assessment scores. The MMSE, which serves as an overall examination of cognition, indicated cognitive decline in the institutionalized elderly, which was supported by the impairment of specific cognitive functions, such as language, semantic memory, and executive function, as evaluated by Verbal Fluency and Boston's Test. Social relations are extremely important for the physical and mental health of the elderly and, unlike the social isolation, which often occurs in the institutionalization process, are some of the most important components of life quality [55]. Cognitive impairment affects the individual's functional capacity in daily life and personal relations and is implied in loss of independence and autonomy, which varies according to severity, resulting in loss of life quality in the elderly [55].

Moreover, the alterations on cognitive function were associated with the oxidative stress and the inflammation markers. Indeed, the central nervous system is particularly vulnerable to oxidative stress due to large rate of oxygen consumption, the abundance of iron, and reduced amounts of antioxidants [56]. The brain is a major metabolizer of oxygen of the body and also contains a large amount of polyunsaturated peroxidizable fatty acids [16]. Therefore, the higher protein damage and the lower activity of GPx may contribute to demyelination and axonal damage, which may represent the underlying cognitive impairment. Such damage is a critical process in the pathogenesis of several chronic∖diseases, but the precise contribution of oxidative stress to age-related cognitive decline remains unclear. According to di Penta et al. (2013), axons and myelin are damaged by both the induction of oxidative stress and release of proinflammatory cytokines, after microglial activation [56].

Lycopene was associated with a better cognitive performance, demonstrating the possible protective action of this micronutrient. Its protective action was also observed by the negative correlation found with the inflammation marker IL-1*β*. Lycopene is the most powerful antioxidant of the carotenoid family [57, 58] and potently prevents lipid peroxidation in synaptic membranes [59], preserving the activity of endogenous free radical scavengers and regulating cholesterol metabolism [58]. The HDL lipoproteins are responsible for the removal of cholesterol and other lipoproteins from peripheral tissues, sending them to the liver for disposal [60]. Therefore, it is important to maintain normal levels of HDL through a balanced diet and avoiding* trans* fats [60]. This corroborates with the correlations found between HDL and the antioxidant micronutrients, lycopene, and vitamin C. Although both study groups have presented normal levels of HDL in this study, it was possible to observe that higher levels of HDL were accompanied by lower PCO levels and by better cognitive performance, showing a protective role of this lipoprotein. In fact, antioxidant and anti-inflammatory activity have been attributed to the HDL that can act reducing the risk of vascular and heart disease [60].

Although the mechanism of age-related cognitive disability is not yet known, it is multifactorial. In this way, age-related inflammatory changes are likely to contribute. High levels of both IL-1*β* and TNF-*α* were shown to be associated with deficit in orientation, memory, attention, and spatial abilities. This finding can be explained in part given that learning and memory processes rely on the hippocampus and this brain region expresses more IL-1 receptors than other regions, making it vulnerable to the negative consequences of neuroinflammation [61]. Thus, the distribution of inflammatory cytokine expression may account, at least in part, for the differential effects on specific cognitive functions, and certain brain regions could be more susceptible to these effects [8]. In this context, it has been described that the long-term maintenance of high IL-1*β* levels, particularly in the hippocampus, may be responsible for hippocampal-dependent memory impairments observed in aging rats [9]. A previous study reported that mice devoid of the cognate receptor to IL-1 and mice given administration of IL-1 receptor antagonist presented significant improvement in cognitive dysfunction [61]. Similarly, elevated TNF-*α* may contribute to a dysregulation of synaptic homeostasis causing short-term recognition and long-term spatial memory deficits, once an agent with anti-TNF-activity, in a model of chronic neuroinflammation restored cognitive function in rats [62], including the hippocampus [62, 63].

Multiple mechanisms have been reported to clarify how the inflammation, especially within CNS, impairs a variety of cognitive domains, for example, by leading to alterations in neuronal function, impaired long-term potentiation, and regulation of gene expression [8], with significant reduction in genes known to be involved in learning and memory, such as the plasticity-related immediate early gene Arc by both IL-1*β* and TNF-*α* [62, 64]. It has also been speculated that, in the brain, cytokines interact with surface's receptors of microglia cells [61]. Upon activation, microglia changes morphologically and secretes cytokines and excitotoxins as well as ROS and neurotoxins, which are able to cause neuronal death [16]. Moreover, neurogenesis in the hippocampus is also inhibited by activated microglia [61], therefore exacerbating the extent of injury on memory processing that is difficult to reverse. Thus, the present findings emphasize that the inflammatory response amplified by cytokines could impact the neuronal functioning.

Although peripheral inflammation may not precisely mirror the situation within the CNS, it has also been described to be involved in producing cognitive dysfunction; thus, the current data are consistent with earlier investigations, which showed the association of serum cytokines with lower cognitive performance [65–67].

Taking into account that both oxidative stress and inflammation can affect the cognitive function, multiple linear regression analyses were conducted. Even though high levels of proinflammatory cytokines have been associated with increased risk of cognitive decline [65], they did not show significant effect. The following confounding factors age and comorbidities also did not present significant influence on cognitive performance. The Boston Naming Test, GPx activity, was found to be the best predictor of cognitive performance, demonstrating the involvement of an endogenous antioxidant. The same was not observed in the case of MMSE and Verbal Fluency, in which the relative influence of educational status was higher. This fact is in agreement with previous studies related to MMSE [27, 68]. The relationship between education and cognitive performance can be explained by the fact that a greater stimulus in different cognitive functions, such as reading, arithmetic, reasoning, abstraction, and planning, leads to the development of higher connectivity among different brain areas and this results in positive effects on the preservation of cognitive functions in old age [69], which may be evaluated by MMSE [27].

Furthermore, the chosen models of multiple regressions corresponded to ~42%, 34%, and 44% of the cognitive performance evaluated by MMSE, Verbal Fluency, and Boston Naming Test, respectively, suggesting that multiple other factors may contribute to the pathophysiology of cognitive decline in the elderly. Nevertheless, this study demonstrated for the first time the association of cytokines and oxidative stress and their impact on cognition in elderly subjects and that, among the studied antioxidants, the endogenous defense appears to be important against cognition loss with respect to orientation, memory, attention, and language skills. Thus, some simple measures that could be implemented, especially in nursing homes, can be crucial in maintaining the health of the elderly, improving their quality of life. Such measures are the regular physical activity, reduction of alcohol consumption and smoking, adequate sun exposure, and mainly change in dietary habits by replacing the* trans* and saturated fats by polyunsaturated and monounsaturated fatty acids found in fish and some oils, respectively, and also increasing the intake of fruits, vegetables and legumes, rich sources of fiber, vitamins, and antioxidants, as lycopene. This study is, however, limited by the use of a brief cognitive screening instrument and small sample size. Future studies should be carried out expanding the number of participants, along with the assessment of cytokine secretion by peripheral blood mononuclear cells and other inflammatory markers, such as adhesion molecules.

In summary, cytokine levels were altered and impaired cognition was observed in the institutionalized elderly group. Neuroinflammation due to age-related overproduction of proinflammatory cytokines has been described as causative factors in development of age-associated neurodegenerative conditions [8]. The cross-sectional nature of this work does not allow drawing any conclusions regarding causation; nonetheless, according to the present findings, oxidative stress seems to be a key contributor to cognitive impairments and lycopene may preserve cognitive functions. Therefore, the elderly with lower antioxidant status were found to be more vulnerable to oxidative stress, which may have negative consequences on the quality and duration of life. At last, although the aging process has yet to be fully understood, oxidative stress damage is a suitable marker of unsuccessful aging.

## Figures and Tables

**Table 1:** Baseline characteristics and prevalence of comorbidities of the studied sample.

Variable	Institutionalized (*n* = 32)	Noninstitutionalized (*n* = 25)
Age, mean (SEM)	78.8 (1.5)^a^	71.2 (1.5)
Years of education, mean (SEM)	4.8 (0.5)^a^	11.1 (0.8)
Gender, frequency (%)		
Male	9 (28.1)	11 (44.0)
Female	23 (71.9)	14 (56.0)
Barthel Index, mean (SEM)	95.4 (1.6)^a^	98.4 (1.4)
Charlson Comorbidity Index, mean (SEM)	3.8 (0.2)^a^	3.2 (0.2)
Comorbidities (%)		
Hypertension	18 (56.2)	18 (72.0)
Diabetes mellitus type 2	6 (18.7)	5 (20.0)
Dyslipidemia	2 (6.2)	6 (24.0)
Osteoporosis	6 (18.7)	1 (4.0)
Hypothyroidism	0 (0.0)	3 (12.0)
Arrhythmia	1 (3.1)	1 (4.0)
Chronic obstructive pulmonary disease	1 (3.1)	2 (8.0)
Gastritis	1 (3.1)	2 (8.0)
Benign prostatic hyperplasia	0 (0.0)	2 (8.0)
Glaucoma	1 (3.1)	1 (4.0)
Chronic renal failure	1 (3.1)	0 (0.0)
Chronic venous insufficiency	1 (3.1)	0 (0.0)
Hyperuricaemia	1 (3.1)	0 (0.0)
Osteoarthritis	1 (3.1)	0 (0.0)
Angina pectoris	1 (3.1)	0 (0.0)

The values are expressed as mean (SEM) or frequency (percentage).

^a^P < 0.05.

**Table 2:** Oxidative status in the studied groups of the elderly.

Biomarkers	Institutionalized (*n* = 32)	Noninstitutionalized (*n* = 25)
MDA (*µ*mol L^−1^)	7.59 ± 0.17^a^	6.22 ± 0.27
PCO (nmol mg^−1^ protein)	0.37 ± 0.01^b^	0.30 ± 0.01
GPx (*µ*mol NADPH min^−1^ mg^−1^ protein)	9.51 ± 0.53^b^	17.31 ± 0.94

The values were adjusted for age and Charlson Comorbidity Index and expressed as mean ± SEM.

^a^ *P* < 0.05.

^b^ *P* < 0.001.

**Table 3:** Exogenous antioxidants in the studied groups of the elderly.

Analyte	Institutionalized (*n* = 32)	Noninstitutionalized (*n* = 25)	Reference value^#^
Lycopene (*µ*mol L^−1^)	0.53 ± 0.04^b^	0.86 ± 0.06	0.4–0.6
*β*-Carotene (*µ*mol L^−1^)	0.62 ± 0.06^a^	0.77 ± 0.07	0.19–1.58
Retinol (*µ*mol L^−1^)	2.41 ± 0.11^b^	3.32 ± 0.16	1.05–2.80
*α*-Tocopherol (*µ*mol L^−1^)	29.70 ± 1.17^b^	37.70 ± 1.79	12–42
Vitamin C (mg L^−1^)	7.35 ± 0.64	7.93 ± 0.78	4–15

The values were adjusted for age and expressed as mean ± SEM.

^#^According to Burtis et al., Tietz Fundamentals of Clinical Chemistry, St. Louis: Saunders/Elsevier, 2008 [32].

^a^ *P* < 0.05.

^b^ *P* < 0.001.

**Table 4:** Correlation coefficients between inflammation markers versus oxidative damage and antioxidant biomarkers (*n* = 57).

Cytokines	MDA (*µ*mol L^−1^)	PCO (nmol mg^−1^ protein)	GPx activity (*µ*mol NADPH min^−1^ mg^−1^ protein)
IL-1*β* (pg mL^−1^)	*r* = 0.444; *P* < 0.01	*r* = 0.369; *P* < 0.01	*r* = −0.546; *P* < 0.001
IL-6 (pg mL^−1^)	*r* = 0.438; *P* < 0.01	*r* = 0.299; *P* < 0.05	*r* = −0.485; *P* < 0.001
TNF-*α* (pg mL^−1^)	*r* = 0.460; *P* < 0.01	*r* = 0.367; *P* < 0.01	*r* = −0.510; *P* < 0.001
IFN-*γ* (*µ*g mL^−1^)	*r* = 0.345; *P* < 0.05	*r* = 0.278; *P* < 0.05	*r* = −0.385; *P* < 0.01

**Table 5:** Cognitive performance of the studied groups of the elderly.

Instrument	Institutionalized (*n* = 30)	Noninstitutionalized (*n* = 22)
MMSE	21.36 (8–29)^b^	27.32 (20–30)
Verbal Fluency	12.14 (2–26)^a^	17.32 (7–31)
Boston Naming Test	10.73 (5–15)^b^	13.91 (9–15)

The values were adjusted for age and Charlson Comorbidity Index and expressed as mean (range).

^a^ *P* < 0.05.

^b^ *P* < 0.01.

**Table 6:** Correlation coefficients among factors involved in oxidative status and cognitive parameters (*n* = 52).

Factors	MMSE	Verbal Fluency	Boston Naming Test
MDA (*µ*mol L^−1^)	*r* = −0.425; *P* < 0.01	*r* = −0.326; *P* < 0.05	*r* = −0.432; *P* < 0.01
PCO (nmol mg^−1^ protein)	*r* = −0.344; *P* < 0.05	*r* = −0.220; *P* > 0.05	*r* = −0.375; *P* < 0.01
GPx (*µ*mol NADPH min^−1^ mg^−1^ protein)	*r* = 0.543; *P* < 0.001	*r* = 0.398; *P* < 0.01	*r* = 0.561; *P* < 0.001
Lycopene (*µ*mol L^−1^)	*r* = 0.157; *P* > 0.05	*r* = 0.221; *P* > 0.05	*r* = 0.388; *P* < 0.01

**Table 7:** Multivariate analysis: factors associated with cognition performance (regression model).

Variable	MMSE *R* ^2^ = 0.424		Verbal Fluency *R* ^2^ = 0.343		Boston Naming Test *R* ^2^ = 0.441	
	*β*	*P* value	*β*	*P* value	*β*	*P* value
GPx (*µ*mol NADPH·min^−1^·mg^−1^ protein)	0.221	0.116	0.218	0.254	0.385	0.029
Lycopene (*µ*mol·L^−1^)	−0.055	0.695	0.153	0.304	0.152	0.265
PCO (nmol·mg^−1^ protein)	−0.104	0.487	0.018	0.906	−0.168	0.242
MDA (*µ*mol L^−1^)	−0.070	0.656	0.024	0.887	0.078	0.609
TNF-*α* (pg·mL^−1^)	0.389	0.337	0.327	0.445	0.121	0.752
IL-1 (pg·mL^−1^)	−0.291	0.474	−0.217	0.613	0.062	0.872
Charlson Comorbidity Index (score)	0.117	0.403	−0.072	0.632	−0.146	0.284
Education (years)	0.532	0.002	0.391	0.030	0.239	0.123

*β*: standardized coefficient beta; *R* ^2^: determination coefficient.
